# Yi Gong San inhibits tumor immune escape by sensitizing colorectal cancer stem cells via the NF-κB pathway

**DOI:** 10.1186/s41065-025-00412-9

**Published:** 2025-04-17

**Authors:** Peng Shen, Shunli Wu, Yi Chen, Guangjing Feng, Xue Guo, Yingguo Chen, Zhigang Wang, Youfeng Shen, Hongbo Wang, Ke Li

**Affiliations:** 1 Department of General Surgery, Chongqing Traditional Chinese Medicine Hospital, Chongqing, 400000 China; 2 Infection Control Department, Chongqing Traditional Chinese Medicine Hospital, Chongqing, 400000 China; 3 Department of Laboratory Medicine, Chongqing Precision Medical Industry Technology Research Institute, Chongqing, China

**Keywords:** Gomisin B, TLR4, Inflammatory factor, Network pharmacology

## Abstract

**Objective:**

Colorectal cancer (CRC), as a highly prevalent malignant tumor globally, faces the dual challenges of drug resistance of cancer stem cells and immune escape in its treatment. Although the traditional Chinese medicine Yigong San (YGS) shows potential in improving the clinical adverse reactions of CRC, its core active components and mechanism of action remain unclear. Based on network pharmacology screening, this study for the first time discovered that Gomisin B might regulate the progression of CRC through the Toll-like receptor 4/Nuclear Factor-kappa B (TLR4/NF-κB) signaling pathway, and aimed to systematically reveal the molecular mechanisms by which YGS and Gomisin B inhibited the malignant phenotypes and immune escape of CRC cells.

**Methods:**

The The Cancer Genome Atlas (TCGA) database was integrated with network pharmacology analysis to screen for the key target of CRC, Gomisin B, and its associated TLR4/NF-κB pathway. Through in vitro CRC stem cell models and mouse xenograft tumor models, techniques such as CCK-8, Transwell, flow cytometry, qPCR/WB were used to evaluate the effects of YGS and Gomisin B on proliferation, migration, invasion, apoptosis, and epithelial-mesenchymal transition (EMT), and to detect the expression of TLR4 and downstream inflammatory factors.

**Results:**

Both YGS and Gomisin B inhibited the proliferation, migration and invasion of CRC stem cells and tumor tissues. Meanwhile, they promoted apoptosis but reduced the expression of the inflammatory factor TLR4 and proteins associated with the NF-κB pathway, thereby exerting suppressive effects on tumorigenesis and disease progression. YGS might also impede EMT progression through modulation of the NF-κB pathway.

**Conclusion:**

This study for the first time elucidated the dual anti-tumor mechanisms of YGS, which sensitized CRC stem cells and inhibited immune escape by targeting the TLR4/NF-κB pathway through Gomisin B. It provides a pharmacological basis for the modern research of traditional Chinese medicine compound against CRC.

**Clinical trial number:**

Not applicable.

**Supplementary Information:**

The online version contains supplementary material available at 10.1186/s41065-025-00412-9.

## Background

Colorectal cancer (CRC), also known as large intestine cancer, ranks among the prevalent malignancies affecting the digestive system. As the third most commonly diagnosed cancer type globally, CRC has approximately 1.9 million new cases each year, and this figure accounts for 10% of all newly diagnosed cancer cases worldwide [1].Alarmingly, the trend of incidence and mortality rates of CRC keeps increasing globally [2]. Metastasis is a relatively common occurrence among patients with CRC, and it is also the primary cause of death for patients with CRC [3]. Therefore, CRC is a significant risk to human well-being and individual life. Recommended options for CRC management mainly include surgery, chemoradiation and drug therapy, but all of them have limitations, such as evident adverse reactions [4] or limited effectiveness in patients with metastatic CRC [5].

In response to the adverse reactions caused by chemotherapy in cancer treatment, traditional Chinese medicine has become a viable treatment option [6]. Yi Gong San (YGS) is a classic formula among traditional Chinese medicines. It is primarily used to enhance physical strength, improve immune function, and regulate the body’s balance. It is often used as an adjuvant treatment for diseases characterized by physical weakness and low immunity. In recent years, some studies have begun to focus on the potential roles of YGS in modern diseases, especially its research and application in cancers, including research on colorectal cancer. YGS primarily controls the release of inflammatory factors via modulating signaling pathways related to inflammation, such as nuclear factor κ-B (NF-κB), as well as its antioxidant action, which has been demonstrated in a variety of animal and cell models of inflammation [7]. The NF-κB signaling pathway is usually in a constitutively activated state in CRC, and this activated state is closely related to the proliferation, survival, invasion, and immune escape of tumor cells [8]. The study has found that curcumin can reduce the expression levels of NF-κB p65 and the apoptosis inhibitor protein survivin in CRC cells, inhibit the phosphorylation of IκBα, increase the expression level of IκBα protein, and further suppress the activation of the NF-κB pathway, so as to prevent and treat CRC [9]. In addition, after treatment with baicalin, the expression levels of Toll-like receptor 4 (TLR4), NF-κB, p65, and p-IκBα in CRC cells were significantly downregulated, and the inhibitory effect was weakened after the use of a TLR4 activator. This indicates that baicalin can inhibit the migration and invasion of CRC cells by impairing the TLR4/NF-κB signaling pathway [10]. Although there is increasing evidence supporting the efficacy of YGS in managing CRC metastasis [11], few studies have explored its efficacy and mechanisms in CRC treatment. Therefore, this study aimed to delve into molecular mechanisms of YGS administration against CRC treatment.

Immune cells and tissue cells can produce and release soluble polypeptides called inflammatory factors. Being intermediaries, these molecules facilitate intracellular communication and regulation, and they play a vital role in body immune response. Interleukin is a class of polymorphic molecules produced by macrophages and T lymphocytes that act on leukocytes and other tissues. They are divided into several families: Interleukin-1 (IL-1), Interleukin-6(IL-6), Interleukin-8 (IL-8), Interleukin-10 (IL-10), and Interleukin-17 (IL-17). Among these, IL-6, IL-8, IL-17 are closely related to CRC [12]. Interleukin-1 beta (IL-1β) has been indicated to be capable of inducing the expression of numerous metastasis-related factors, thereby promoting tumor growth and metastasis. Likewise, IL-8 contributes to tumor progression and metastasis by involving in angiogenesis, supporting cancer stem cells, recruiting neutrophils, and driving the epithelial-mesenchymal transition (EMT) of tumor cells [13]. As an important inflammatory medium, Tumor Necrosis Factor-alpha (TNF-α) serves as a crucial component in stimulating inflammatory cells, maintaining cellular homeostasis, and promoting tumor development [14]. This research sought to elucidate the impact of YGS on the development of CRC through the previously described indicators. The process of EMT is vital for encouraging the invasion of cancer cells with great significance in the progression of CRC [15]. Within the EMT process, there are multiple changes in biomarkers, including the suppression of E-cadherin and keratin expression, and the elevation of expression of interstitial cell marker proteins vimentin, fibronectin, and N-cadherin [16]. Understanding the mechanism of EMT changes associated with YGS in CRC provides potential for targeted therapy of CRC.

Network pharmacology, a new mode that mines and studies the active ingredients of drugs based on big data analysis, when organically combined with traditional Chinese medicine, has become an innovative idea and an effective approach for in-depth research on the action mechanisms of traditional Chinese medicine compounds [17]. Through the combination of network pharmacology and in vivo and in vitro experiments, this study found that Gomisin B in YGS may target Toll-like receptor 4 (TLR4), and sensitize CRC stem cells via the NF-κB signaling pathway, inhibit tumor immune escape, and suppress the EMT process. Thus, this study systematically elucidates the molecular mechanism of YGS in the treatment of CRC, offering promising new ideas and methods for the treatment of CRC.

## Materials and methods

### YGS preparation

The formula originated from “Key to Therapeutics of Children’s Diseases” ([18] and the ingredients included *Panax ginseng C. A. Mey.* (Reed removed) 9 g, *Atractylodis Macrocephalae Rhizoma* 9 g, peeled *Poria cocos (Schw.) Wolf.* 9 g, processed *Glycyrrhizae.* 6 g, and *Citrus L.* 6 g (Zesam Pharmacy). The ingredients were soaked in water for 1 h in a volume ratio of 1:7, boiled for 30 min and filtered. The dregs were boiled in water for 20 min in a volume ratio of 1:3 and filtered. We combined the filtrates obtained from the two boilings, and placed the combined filtrate in a rotary evaporator. We concentrated it under the conditions of a temperature of 60℃ and a pressure of -0.08 MPa until the concentrated liquid was equivalent to containing 2 g of medicinal herbs per milliliter. During the preparation process, we used deionized water for soaking and boiling to ensure that we could make the components of the medicinal herbs fully dissolved. The final concentrated liquid was stored at 4℃ for future use.

### CRC stem cell isolation and identification

SW480 CRC cell line (CVCL: 0546) was purchased from Wuhan Pricella Biotechnology Co., Ltd. (CL-0223B). CRC stem cells were isolated from the SW480 CRC cell line using magnetic beads [19]. The CRC stem cells were collected and washed once with Phosphate Buffered Saline (PBS), and a DMEM/F12 medium (L210KJ, BasalMedia, Shanghai, China) containing Epidermal Growth Factor (EGF, 45086ES60, Yeasen, Shanghai, China), Basic Fibroblast Growth Factor (FGF-basic, HY-P7331, MCE, NJ, USA), B-27 (60703ES10, Yeasen, Shanghai, China), and Antibiotic-Antimycotic (orb640253, Biorbyt, Cambridge, UK) without fetal bovine serum was added, and then they were cultured in ultra-low adhesive 6-well plates. The culture conditions were 37℃, 5% CO_2_, and saturated humidity. The mice were categorized into four groups: control (no treatment), YGS low-dosage (15 g/(kg·d)), YGS medium-dosage (20 g/(kg·d)), and YGS high-dosage groups (25 g/(kg·d)). After 7 d of gavage, the mice were euthanized with 0.3% sodium pentobarbital for serum sample collection. Subsequently, the serum was employed to treat CRC stem cells, which were categorized into the following groups: control, YGS low dose, YGS medium dose, and YGS high dose. Samples for testing were collected 48 h later.

CRC stem cells were divided into a CRC stem cell group, stem cell + YGS (1 mg/mL) group, stem cell + Gomisin B (10 µM, PHL83542, Macklin, Shanghai, China) group, and stem cell + Gomisin B + TLR4 overexpression group. After 48 h, CCK-8, cell scratch, Transwell invasion and flow cytometry were performed.

### Establishment of nude mouse model with transplanted CRC cells

Twenty-four male Specific Pathogen Free (SPF)-grade nude mice were supplied by Chongqing Ensiweier Biotechnology Co. Ltd., each at six weeks of age and weighing 20 g. All these mice were housed under identical conditions. The experiment was commenced after one-week adaptive feeding. The animal models of CRC graft tumors were divided into a CRC stem cell group (intraperitoneal injection of a certain volume of saline), cisplatin (intraperitoneal injection 5 mg/kg) + stem cell group, heterodynamic dispersion (gavage 25 g/(kg d)) + cisplatin + stem cell group, and Gomisin B (intraperitoneal injection 20 mg/kg) + cisplatin + stem cell group. Tumor stem cells were inoculated with 200 µL (about 1 × 10^4^ cells/mouse) subcutaneously in the right axilla of nude mice and their status was observed 7 d later.

This animal research was scrutinized and granted approval by the Ethics Committee of Chongqing Hospital of Traditional Chinese Medicine (No. 2021-ky-46), and strictly adhered to the animal ethics procedures and guidelines. In this study, we strictly monitored the tumor size of experimental animals and confirmed that it did not exceed the maximum tumor size permitted by the ethics committee.

### Screening of CRC hub genes

This study screened 80 CRC samples utilizing data from The Cancer Genome Atlas (TCGA) database (https://portal.gdc.cancer.gov/), which were assigned to the experimental group and the control group respectively, with 40 samples each. Differentially Expressed Genes (DEGs) were analyzed using the R package DEseq2. The main components of different genes were analyzed by gmodels package to observe the discrete and clustering patterns. Visualization modules for module-module clustering and trait-module clustering were established employing the weighted gene co-expression network analysis (WGCNA). To further analyze the screened genes, we utilized the David database (https://metascape.org/gp/index.html#/main/step1) to utilize data from Gene Ontology (GO) and the Kyoto Encyclopedia of Genes and Genomes (KEGG) analyses. Subsequently, a diagram of protein-protein interaction network was plotted and exported from String database (https://cn.string-db.org/). Hub genes were pinpointed according to the network topology. Ultimately, the screened genes were used for survival analysis using R package.

### Network pharmacological analysis

YGS components were systematically retrieved from Traditional Chinese Medicine Systems Pharmacology Database and Analysis Platform (TCMSP) database, and potential monomers associated with YGS treatment were obtained. Subsequently, drug target IDs from Universal Protein Resource (UniProt) database (https://www.uniprot.org/) were converted into their corresponding gene terms as cataloged in the GeneCards database (https://www.genecards.org/). We used “CRC” as a keyword to filter disease targets and obtained CRC-related genes from both GeneCards and the Online Mendelian Inheritance in Man (OMIM) database. By cross-referencing these CRC-related genes with the YGS target genes, we identified common targets and determined the action targets of YGS in treating CRC. A network of ingredients-targets-associated disease was constructed based on candidate chemical components and CRC targets through Cytoscape 3.7.2. A PPI diagram was plotted, which had the highest confidence coefficient (> 0.9), by uploading genes to the online String database. GO functional enrichment and KEGG analyses of the proteins were conducted utilizing the DAVID database.

### Detection of cell proliferation ability by CCK-8 assays

The logarithmic cells were washed twice with PBS (B220KJ, BasalMedia, Shanghai, China). After digestion with pancreatic enzyme (S310KJ, BasalMedia, Shanghai, China) and centrifugation, the cell density was adjusted to 1 × 10^4^ cells/well in 96-well plates and cultured for 24 h, and CCK-8 solution (C0038, Beyotime, Shanghai, China) was added for incubation. The optical density at 450 nm was ascertained employing a spectrophotometer (NanoDrop One/OneC, Thermo Fisher Scientific, Waltham, MA, USA).

### Scratch test detection

Straight lines were drawn on the back of 6-well plates with a histochemical pen, crossing three lines per well. Cells at the logarithmic growth stage were seeded in culture plates at a concentration of 5 × 10^5^/mL; After the cells reached 90% confluency, 1 mL of PBS was added to each well to wash the cells three times. Subsequently, a 200 µL pipette was used to scratch the culture plates evenly with force; following PBS washing twice, the scratched cells were removed; 2 mL of conditioned media was added to each well. After 48 h, the distance of cell movement was photographed under a microscope, and Image J was used to count the scratched area based on the formula: cell mobility = (0 h scratch area - post-culture scratch area)/0 h scratch area.

### Transwell invasion detection

Serum-free medium (C11995500BT, Gibco, CA, USA) was evenly mixed with Matrigel (5:1) (356234, Thermo Fisher Scientific, Waltham, MA, USA). After the addition of the mixture to the upper Transwell chamber, 5 × 10^5^ cells resuspended by a serum-free medium were inoculated. A conditioned medium was added to the chamber at 37℃ and cultured in a 5% CO_2_ incubator. Following two cycles of PBS washing, the cells were fixed using 4% polyformaldehyde, dyed by 0.1% crystal violet, washed three times with PBS, and observed using a microscope.

### Flow cytometry apoptosis detection

Cells were cultured in 24-well plates and allowed to grow until reaching 60–70% confluency. The cells were processed according to the respective groups before another culture. The culture medium was then removed. Following treatments based on grouping, a fresh medium was added, and the cells were prepared for analyses. The cells were digested with pancreatic enzyme (S310KJ, BasalMedia, Shanghai, China), transferred to centrifuge tubes, centrifuged, collected, resuspended, and then the cell number was counted. An AnnexinV-FITC-A binding solution (C1056, Beyotime, Shanghai, China) was added to suspend the cells gently. Then AnnexinV-FITC was added, and the mixture was mixed evenly with care and permitted to incubate. The cells were centrifuged to remove the supernatant, the cells were added to which 190 µL of AnnexinV-FITC-A binding solution, resuspended and then 10 µL of PI staining solution was added. After being gently mixed, the samples were placed on ice in the dark for subsequent flow cytometry (CytoFLEX, Beckman Coulter, CA, USA) analyses.

### ELISA

Detection was carried out according to the instructions for use of the Mouse Interleukin 1β (IL-1β) Kit (RX302869R, ruixinbio, Quanzhou, China), the Mouse Interleukin 8 (IL-8) Kit (F2123-A, Fankew, Shanghai, China), and the Mouse Tumor Necrosis Factor alpha (TNF-α) Kit (RX202412M, ruixinbio, Quanzhou, China).

### qPCR

The cells/ground tissues in liquid nitrogen were washed with PBS (G0002, Servicebio, Wuhan, China), then Trizol and the corresponding number of steel beads were added, the mixture was fully oscillated, chloroform (Chuandong Chemical, Chongqing, China) was added, and the mixture was centrifuged. An equal volume of isopropanol was added to the supernatant to precipitate RNA, and then the mixture was centrifuged. The samples were dried and added with Diethylpyrocarbonate (DEPC) water to dissolve RNA. Reverse transcription was performed in accordance with the instructions of Goldenstar™ RT6 cDNA Synthesis Kit (TSK302M, Beijing, China). qPCR was performed using a real-time fluorescence quantitative PCR instrument. Each group had 3 multiple wells with GAPDH as an internal reference. The mRNA level of the indicators of each group was expressed by CT values, which were calculated based on the formula 2^-ΔΔCT^. Primers are displayed in Table 1.

**Table 1:** Primer sequences

Primers	Sequences
h-TLR4-F	TGCGTGGAGGTGGTTCCTA
h-TLR4-R	AGAGGTGGCTTAGGCTCTGA
h-TNF-α-F	TGCACTTTGGAGTGATCGGC
h-TNF-α-R	ACTCGGGGTTCGAGAAGATG
h-IL-1β-F	AGCTCGCCAGTGAAATGATG
h-IL-1β-R	CCTTGCTGTAGTGGTGGTCG
h-IL-8-F	ACTCCAAACCTTTCCACCCC
h-IL-8-R	ATGAATTCTCAGCCCTCTTCAA
h-GAPDH-F	TCAAGGCTGAGAACGGGAAG
h-GAPDH-R	TCGCCCCACTTGATTTTGGA
m-TLR4-F	CACTGTTCTTCTCCTGCCTGA
m-TLR4-R	AGGGACTTTGCTGAGTTTCTGA
m-TNF-α-F	CCACCACGCTCTTCTGTCTA
m-TNF-α-R	GTTTGCTACGACGTGGGC
m-IL-1β-F	ATGCCACCTTTTGACAGTGAT
m-IL-1β-R	AGCCCTTCATCTTTTGGGGT
m-IL-8-F	ATTTCCACCGGCAATGAAGC
m-IL-8-R	GTCTCCCGAATTGGAAAGGGA
m-GAPDH-F	GGAGAGTGTTTCCTCGTCCC
m-GAPDH-R	TTTGCCGTGAGTGGAGTCAT

### WB

The WB test was performed by referring to the approach of Zhang et al. [20]. After being washed with PBS, 1 mL Ripa lysate (P0013B, Beyotime, Shanghai, China) was added to the cells/ground tissues in liquid nitrogen, and then they were centrifuged. Subsequently, 5 × Sodium Dodecyl Sulfate-Polyacrylamide Gel Electrophoresis (SDS-PAGE) protein loading buffer (8015011, Dakewei, Shenzhen, China) was added to the supernatant at a 4:1 rate, and the mixture was denatured in a 100℃ metal bath for 6 min. The loading volume was 60 µg. SDS-PAGE electrophoresis was performed with concentration gel and 12% separation glue for protein separation. The electrophoresis condition was set at 60 V for 30 min, and then 120 V for 60 min until the prestained Marker (8011031, Dakewei, Shenzhen, China) bands were separated. The SDS-PAGE gel was taken out. A Polyvinylidene Fluoride (PVDF) membrane (10600023, Amersham, Aylesbury, UK) was prepared, trimmed to an appropriate size, activated in methanol (201809, Chuandong Chemical, Chongqing, China) for a few seconds, and then laminated with the gel. Three layers of filter paper were placed on each side, wetted with electrolyte. The air was evacuated, then it was tightly clamped and put into an electrolytic transfer tank. The condition was set at 250 mA for membrane transfer, and the PVDF membrane was subsequently soaked in a prepared skim milk (MBchem Company), sealed and gently vibrated on a shaker for 1 h; Antibodies were diluted to an appropriate rate as per antibody instructions, placed with the PVDF membrane and added a primary antibody (1:1000) to an incubation kit, and placed into a thermostatic shaker at 4℃ overnight; The PVDF membrane was removed the next day and rinsed with a Tris Buffered Saline Tween-20 (TBST) (110452, Monad, Suzhou, China) solution. Then the membrane was incubated with mild vibration for 1 h in an incubator, and added with a secondary antibody (antibody: milk = 1:2000). TBST was used for rinsing 3 times; The Enhanced Chemiluminescence (ECL) exposure solution (34580, Thermo Fisher Scientific, Waltham, MA, USA) was added and kept for 1 min, and the PVDF membrane was imaged using an imaging system (Universal Hood II, Bio-Rad Laboratories, Hercules, CA, USA); The GAPDH grayscale values were used as a reference. Each sample was repeated three times.

Antibody information is listed as following: ALDH (RRID: AB_2861455, A0157, abclonal, Wuhan, China), SOX-2 (RRID: AB_2716820, A0561, abclonal, Wuhan, China), LGR5 (RRID: AB_2758086, A10545, abclonal, Wuhan, China), TLR4 (RRID: AB_2766084, A5258, abclonal, Wuhan, China), p-IKKα/β (RRID: AB_2771197, AP0546, abclonal, Wuhan, China), p-IκBα (RRID: AB_2863811, AP0707, abclonal, Wuhan, China), IKKα (RRID: AB_2862739, A19694, abclonal, Wuhan, China), IκBα (RRID: AB_2758831, A1187, abclonal, Wuhan, China), GAPDH (RRID: AB_2862549, A19056, abclonal, Wuhan, China), and secondary antibody (RRID: AB_2769854, AS014, abclonal, Wuhan, China).

### TUNEL staining

A small circle was drawn 2–3 mm away from the tissue outline. Then, 20 µg/mL of Proteinase K was added by drops. The sample was incubated and rinsed with PBS. Subsequently, 3% H_2_O_2_ (prepared with PBS) was added by drops to thoroughly permeate the tissue. After the tissue was rinsed with PBS, Equilibration Buffer was dripped onto the sample for 10 min incubation. Following this, an appropriate amount of Terminal-deoxynucleotidyl Transferase (TdT) (G1507, Servicebio, Wuhan, China) incubation buffer was added. The sample was incubated for 1 h away from light and then washed with PBS four times. A streptavidin-HRP reaction solution was added dropwise, and then rinsed with PBS. An appropriate amount of Diaminobenzidine (DAB) solution (ZSGB-BIO, ZLI-9019) was added for color development. When the result showed positive, the reaction was terminated by washing with pure water. Sulforaphane dye (Servicebio, G1004) was applied for staining for 1 min, and then Sulforaphane differentiation liquid was applied for approximately 2 s. The sample was thoroughly rinsed with pure water and the sections were sealed and subsequently observed using an inverted microscope (MF53, Mshot, Guangzhou, China). The apoptosis rate (%) was computed based on the formula (apoptotic cells/total cells) × 100%.

### Immunohistochemistry

Mouse tumor samples were preserved, followed by paraffin embedding, sectioning, deparaffinization, and rehydration. Subsequently, the specimens were immersed in antigen retrieval solution (G1201, Servicebio, Wuhan, China) for antigen retrieval. After washing with PBS, the sections were incubated with goat serum (C0265, Beyotime, Shanghai, China). The sections were incubated with the primary antibody (LGR5, dilution ratio 1:200, A10545, ABclonal, Wuhan, China) overnight at 4 °C to specifically recognize the target antigen. After washing the specimens with PBS, the sections were incubated with the secondary antibody (dilution ratio 1:200, 511203, Zenbio, Chengdu, China) working solution to amplify the signal from the primary antibody. Following this, the sections were developed with DAB (ZLI-9019, Zsbio, Beijing, China), counterstained with hematoxylin (G1004, Servicebio, Wuhan, China), and subjected to an ethanol gradient dehydration process before being mounted for microscopic examination. The interpretation of staining results was done by observing and photographing under a microscope and performing grayscale value analysis.

### Immunofluorescence

Paraffin slices were dewaxed in different concentrations of xylene (Chuandong Chemical, Chongqing, China), soaked in gradient ethanol (Chuandong Chemical, Chongqing, China), rehydrated and washed three times. The antigen was boiled, retrieved for 30 min using the antigen retrieval solution (G1201, Servicebio, Wuhan, China) and washed three times with PBS after cooling. Following blocking for 30 min with goat serum, the primary antibody was added for incubation (dilution ratio 1: 200) at 4℃overnight, followed by three cycles of PBS washing. Secondary antibody was added for incubation, and then washed three times with PBS. DAPI (C1005, Beyotime, Shanghai, China) was added for incubation and nucleus staining. Following PBS rinse, the sections were sealed with an anti-fluorescent quenching agent (P0126, Beyotime, Shanghai, China). Histological changes were observed under an inverted microscope.

Antibody information is listed as follows: Vimentin Rabbit mAb (R22775, Zenbio, Research Triangle Park, NC, USA), E Cadherin Rabbit pAb (340341, Zenbio, Research Triangle Park, NC, USA), Goat Anti-Rabbit IgG H&L (FITC) (511201, Zenbio, Research Triangle Park, NC, USA), Goat Anti-Rabbit IgG H&L (Cy3) (550076, Zenbio, Research Triangle Park, NC, USA).

### Statistical analysis

Representative results were selected from at least 3 experimental repeats. SPSS 23.0 was applied for data analyses. All results were presented in mean ± standard deviation. Pairwise comparison of two samples was analyzed using unpaired *t*-tests. Multigroup sample comparison was analyzed using one-way ANOVA. *P* < 0.05 was considered to be statistically meaningful.

## Results

### Influences of YGS on metastatic relapse of CRC stem cells

**Fig. 1:**
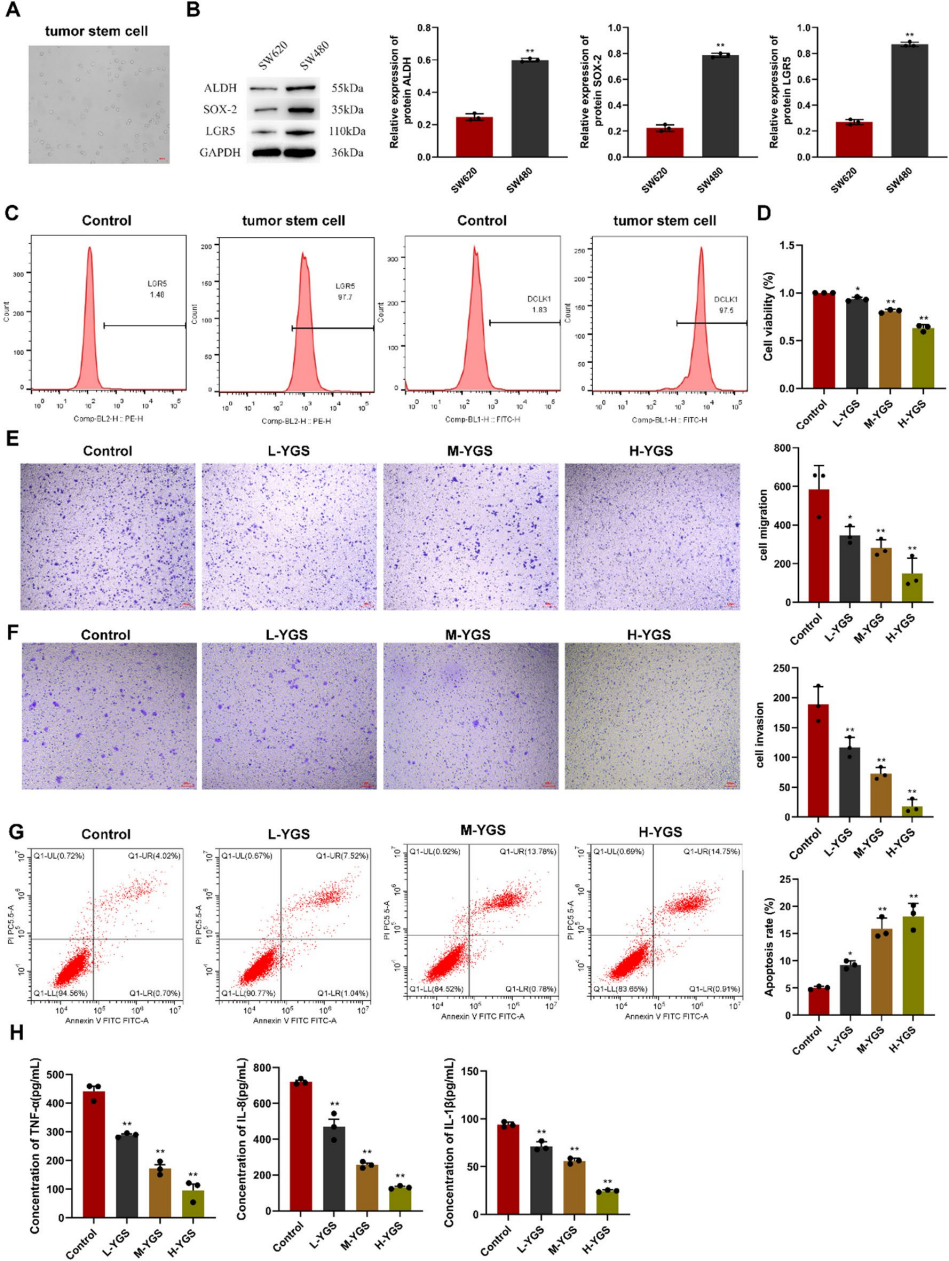
CRC stem cell collection and the effect of YGS on metastatic relapse. **A**, SW480 CRC cell line culture; **B**, Western blot analysis from representative CRC stem cell populations using indicated antibodies; **C**, Identification of SW480 CRC cell line using flow cytometry; **D**, The effect of YGS on the proliferation of CRC stem cells was investigated using the CCK8 assay; **E**, The effect of YGS on the migration ability of CRC stem cells was explored by using the Transwell chamber assay; **F**, The effect of YGS on the invasion ability of CRC stem cells was studied by using the Transwell chamber assay; G, The effect of YGS on the apoptosis of CRC stem cells was investigated by using flow cytometry; **H**, The effect of YGS on the expression of cytokines in CRC stem cells was explored by using ELISA kits. * indicates *P* < 0.05, ** indicates *P* < 0.01, *n* = 3

The morphology of SW480 CRC cells is shown in Fig. 1A. CRC stem cells were separated using magnetic beads, and the contents of ALDH, SOX-2 and LGR5 were detected using WB (Fig. 1B-C). The results showed that the protein contents of ALDH, SOX-2 and LGR5 in SW480 CRC cells were significantly higher than those in SW620 CRC cells (*P* < 0.01), indicating that the CRC stem cells were successfully collected.

Figure 1D presents the results of CCK-8 assays. As the concentration of YGS increased, inhibitory effects on cell proliferation became more pronounced. Figure 1E and F illustrate that as the concentration of YGS increased, the migration and invasiveness of CRC stem cells diminished (*P* < 0.01). Flow cytometric analysis in Fig. 1G reveals that, compared to the control group, the viability of isolated cells decreased as YGS concentration increased, accompanied by a gradual increase in apoptotic cells.

Figure 1H depicts ELISA outcomes. Evidently, with the increasing concentration of YGS, serum levels of TNF-α, IL-8, and IL-1β diminished markedly (*P* < 0.01) when compared to the control group.

### Screening of CRC key genes

**Fig. 2:**
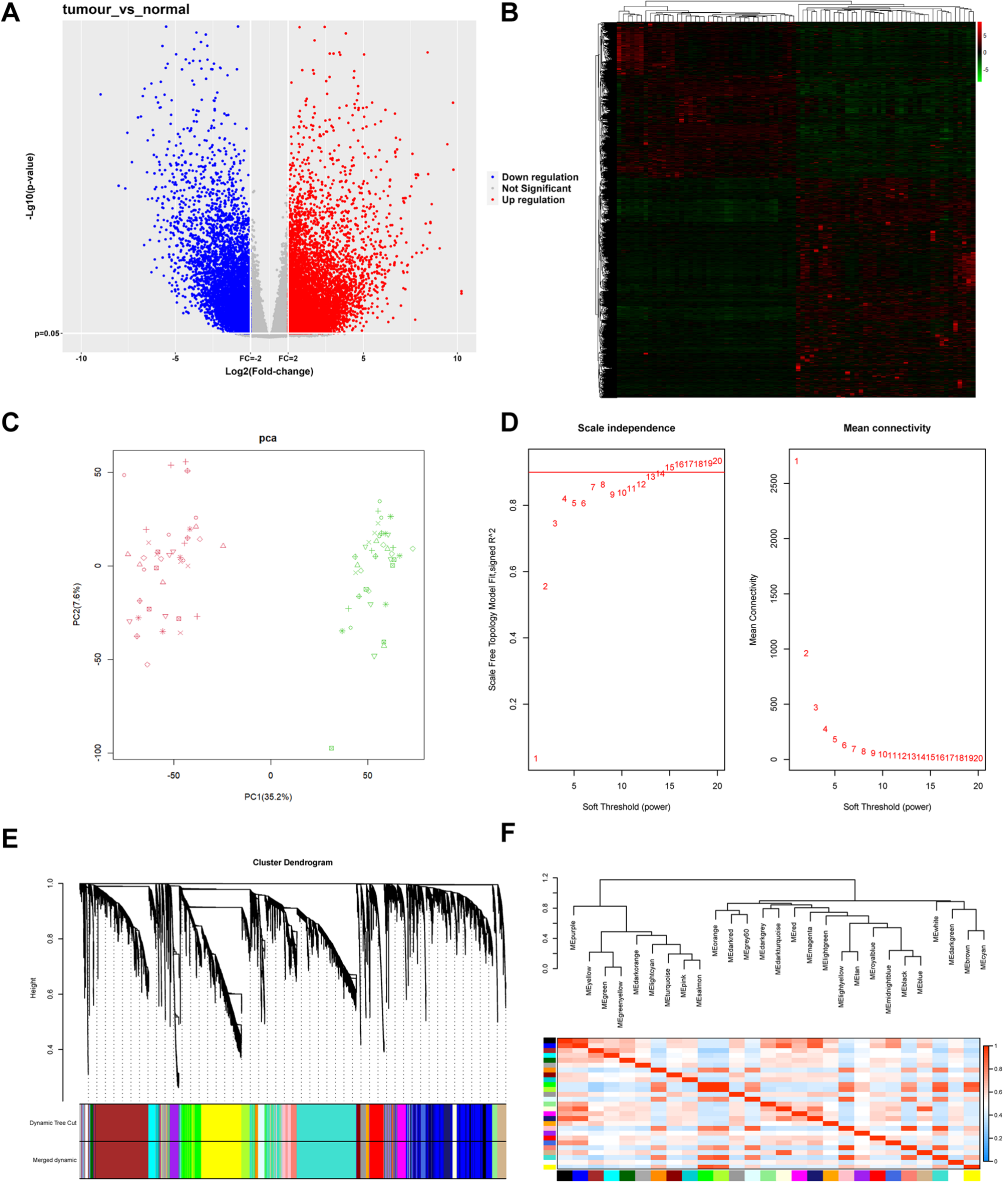
CRC differential gene analysis rooted in the TCGA database. **A**, Volcano map; **B**, Heatmap; **C**, Principal component analysis; **D**, Differential gene WGCNA analysis; **E**, Module clustering of differential genes; **F**, Module visualization of the differential genes

**Fig. 3:**
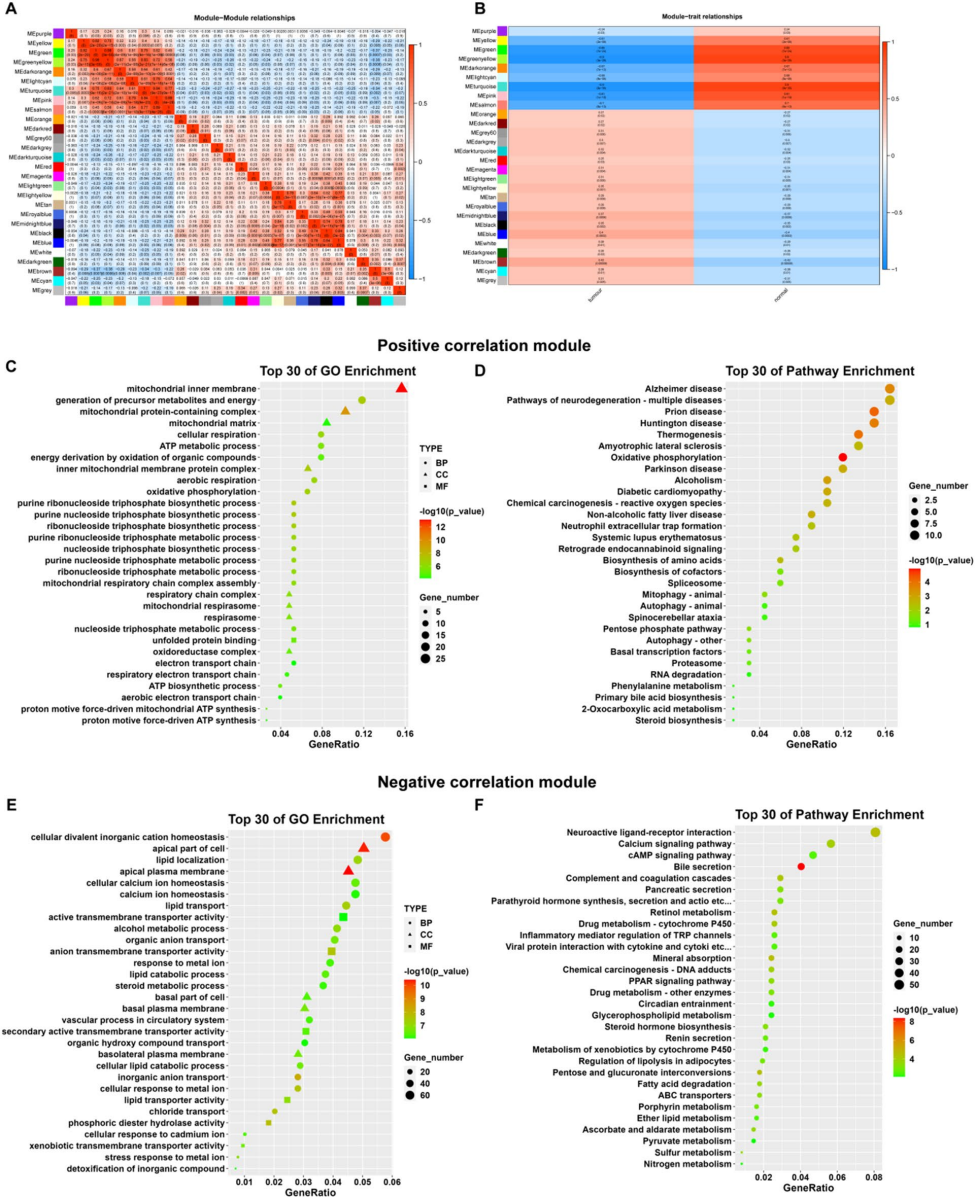
Differential gene module clustering. **A**, Module-module clustering; **B**, Character-module clustering. **C**, Bubble diagram of GO analysis of positively associated differential genes; **D**, Bubble diagram of KEGG analysis of positively associated differential genes. **E**, Bubble diagram of GO analysis of negatively associated differential genes; **F**, Bubble diagram of KEGG analysis of negatively associated differential genes

A total of 13,693 CRC-related differential genes were screened in the CRC and control samples based on the TCGA database, of which 7,922 were up-regulated and 5,771 were down-regulated. The volcano map and heat map are shown in Fig. 2A and B. PCA analysis of DEGs (Fig. 2C) revealed a satisfactory dispersion. WGCNA analysis was also performed with the corresponding soft threshold at 14 and correlation coefficient at 0.85 (Fig. 2D). DEGs were clustered into different gene modules, as shown in Fig. 2E-F. Module-module clustering analysis is shown in Fig. 3A, classifying genes according to expression patterns. Trait-module cluster analysis is shown in Fig. 3B. Some gene modules had a positive link to normal group’s clinical features, and some correlated positively with those of the tumor group.

Based on the WGCNA results, GO and KEGG enrichment analyses of positive and negative correlated module differential genes were performed using the David database to screen out biological processes and signaling pathways of statistical significance, as shown in Fig. 3C-F.

Positively correlated modular genes were enriched for GO biological processes, and 2,214 GO entries were screened on False Discovery Rate (FDR) < 0.05, and the GO analysis results were partially displayed according to degrees of significance. The targets were mainly concentrated in the generation of precursor metabolites and energy, unfolded protein binding, the mitochondrial inner membrane. KEGG analyses revealed that pathways involved included Oxidative phosphorylation, Prion disease, and Thermogenesis.

Negatively correlated modular genes were subjected to GO and KEGG analyses, as shown in Fig. 3E-F. A total of 6,930 GO entries were screened based on FDR < 0.05, and GO analysis results were partially displayed according to degrees of significance. Targets were mainly concentrated in cellular divalent inorganic cation homeostasis, active transmembrane transporter activity, apical part of cell. KEGG analyses revealed that pathways involved included the calcium pathway, cAMP pathway, and PPAR pathway.

**Fig. 4:**
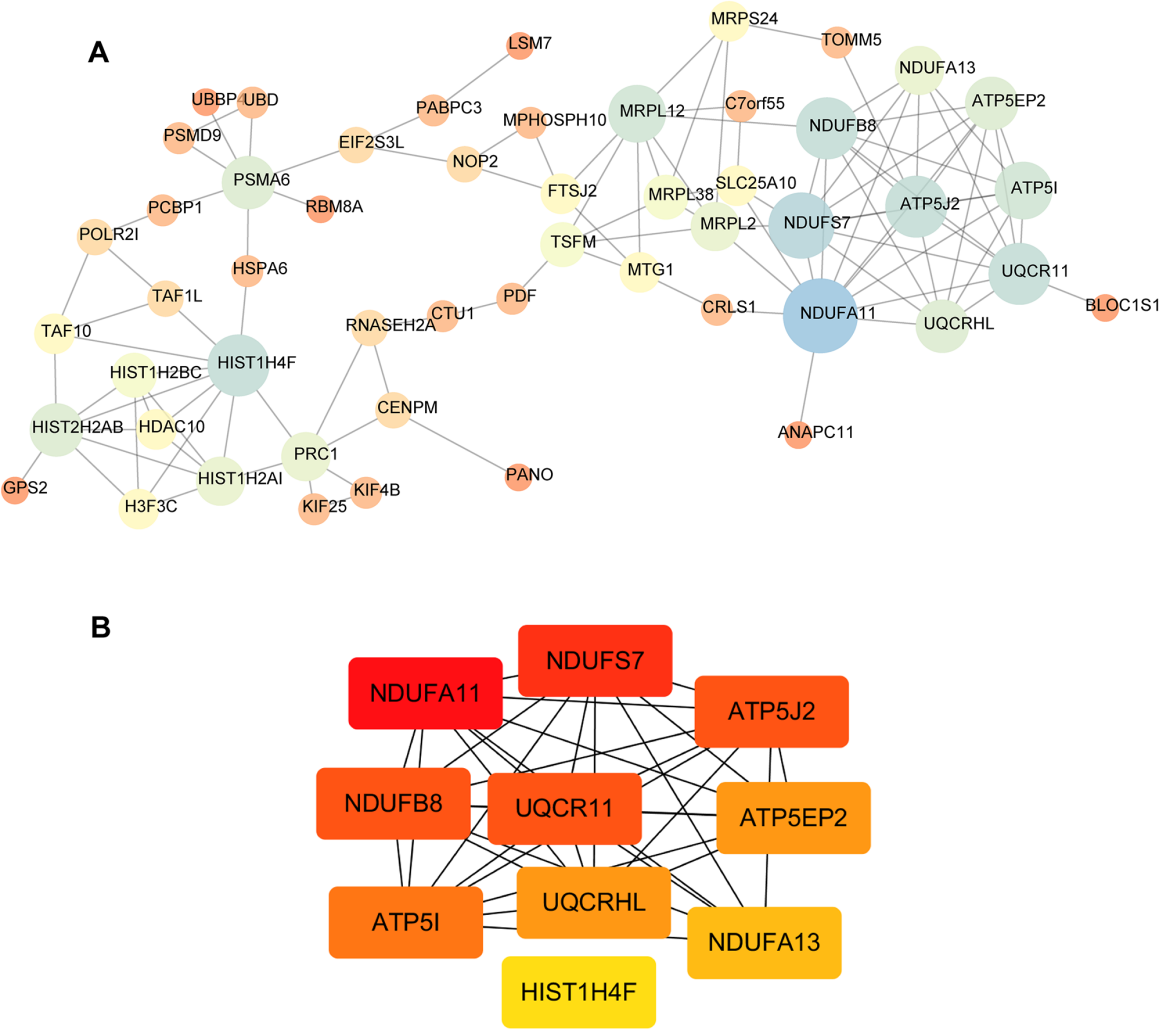
Positive correlation module differential gene interaction analysis. **A**, PPI; **B**, Network topological structure

**Fig. 5:**
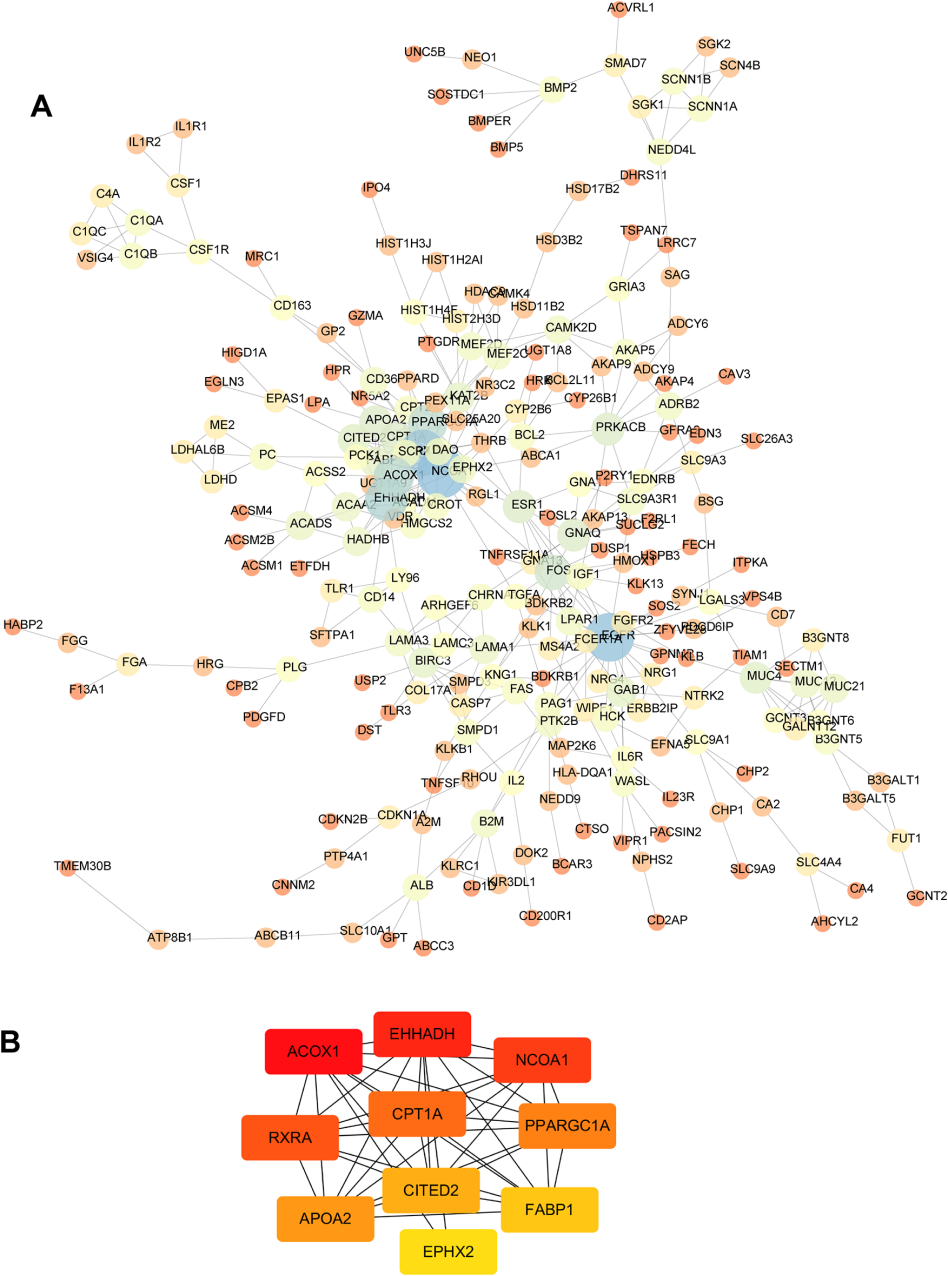
Negative correlation module differential gene interaction analysis. **A**, PPI; **B**, Network topological structure

Based on the WGCNA results, a Protein-Protein Interaction (PPI) network of the obtained DEGs was exported from the String, and hub genes were screened according to network topology, as shown in Figs. 4 and 5. The hub genes were sorted out using the MCC method in the positive correlation module of WGCNA and the top 10 were NDUFS7, NDUFS8, NDUFA11, NDUFA13, ATP5J2, ATP5I, ATP5EP2, UQCR11, UQCRHL, and HIST1H4F; The top 10 hub genes screened by the MCC method in the negative correlation module were EHHADH, ACOX1, NCOA1, RXRA, CPT1A, PPARGC1A, APOA2, CITED2, FABP1, and EPHX2.

### Network pharmacology screening YGS affects candidate monomers target genes and pathway results of CRC stem cell metastasis relapse

**Fig. 6:**
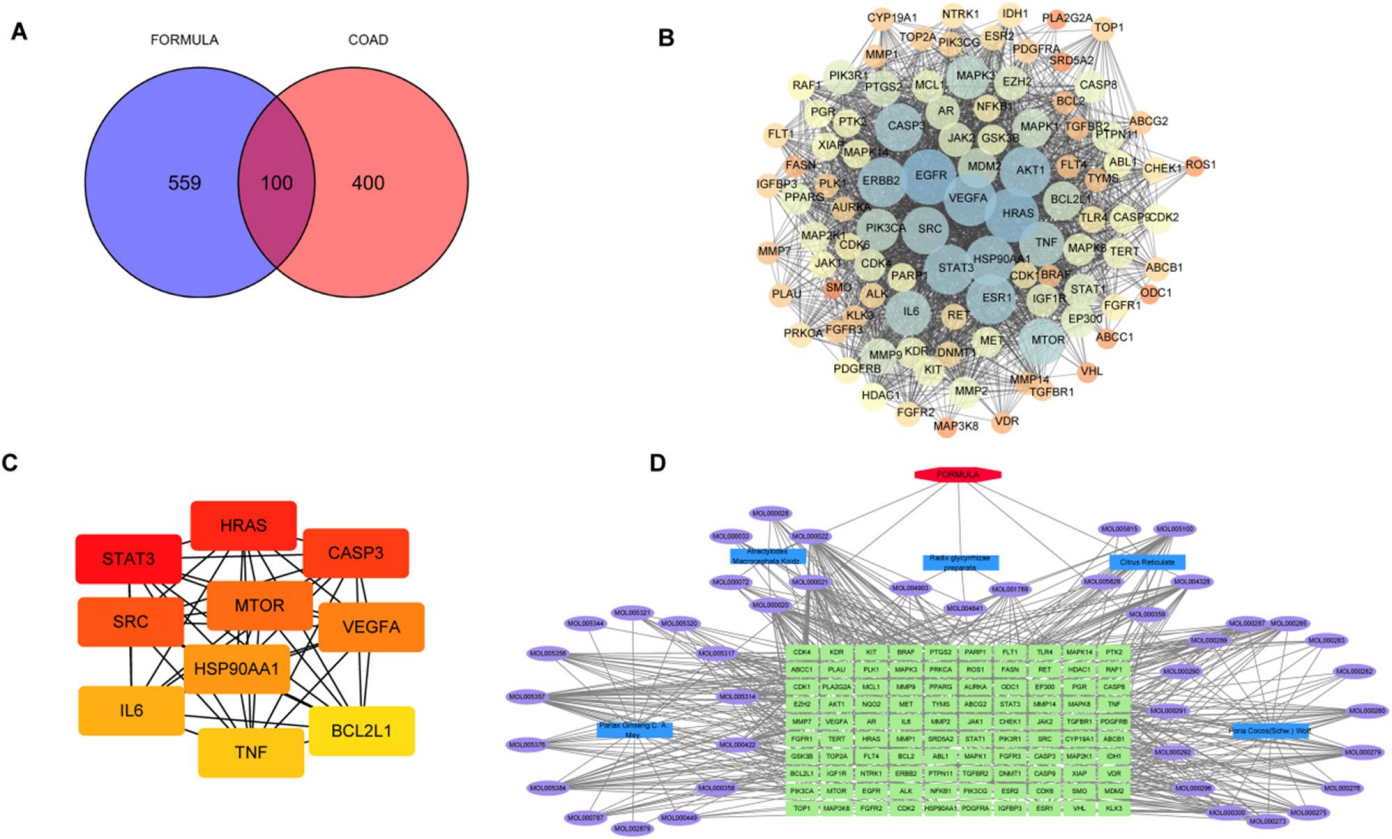
YGS interacts with CRC co-target proteins. **A**, Venn chart; **B**, PPI network of YGS-CRC target protein interaction; **C**, MCC algorithm screening of the top 10 hub genes; **D**, YGS-Target-CRC network

A total of 659 YGS targets were obtained from the TCMSP database through screening, and differential CRC targets were obtained as described in Sect. 3.2, and then the top 500 targets were selected based on the correlation coefficient, and a Venn diagram was plotted and 100 overlapped targets were obtained, as shown in Fig. 6A. The interaction of overlapped target proteins was constructed through the String database, as shown in Fig. 6B. According to the scores of the MCC algorithm, the top 10 genes were STAT3, HRAS, CASP3, SRC, MTOR, VEGFA, HSP90AA1, IL6, TNF, and BCL2L1, as shown in Fig. 6C. The TCM-Core gene-cancer network was constructed using Cytoscape 3.7.2 package, as shown in Fig. 6D.

**Fig. 7:**
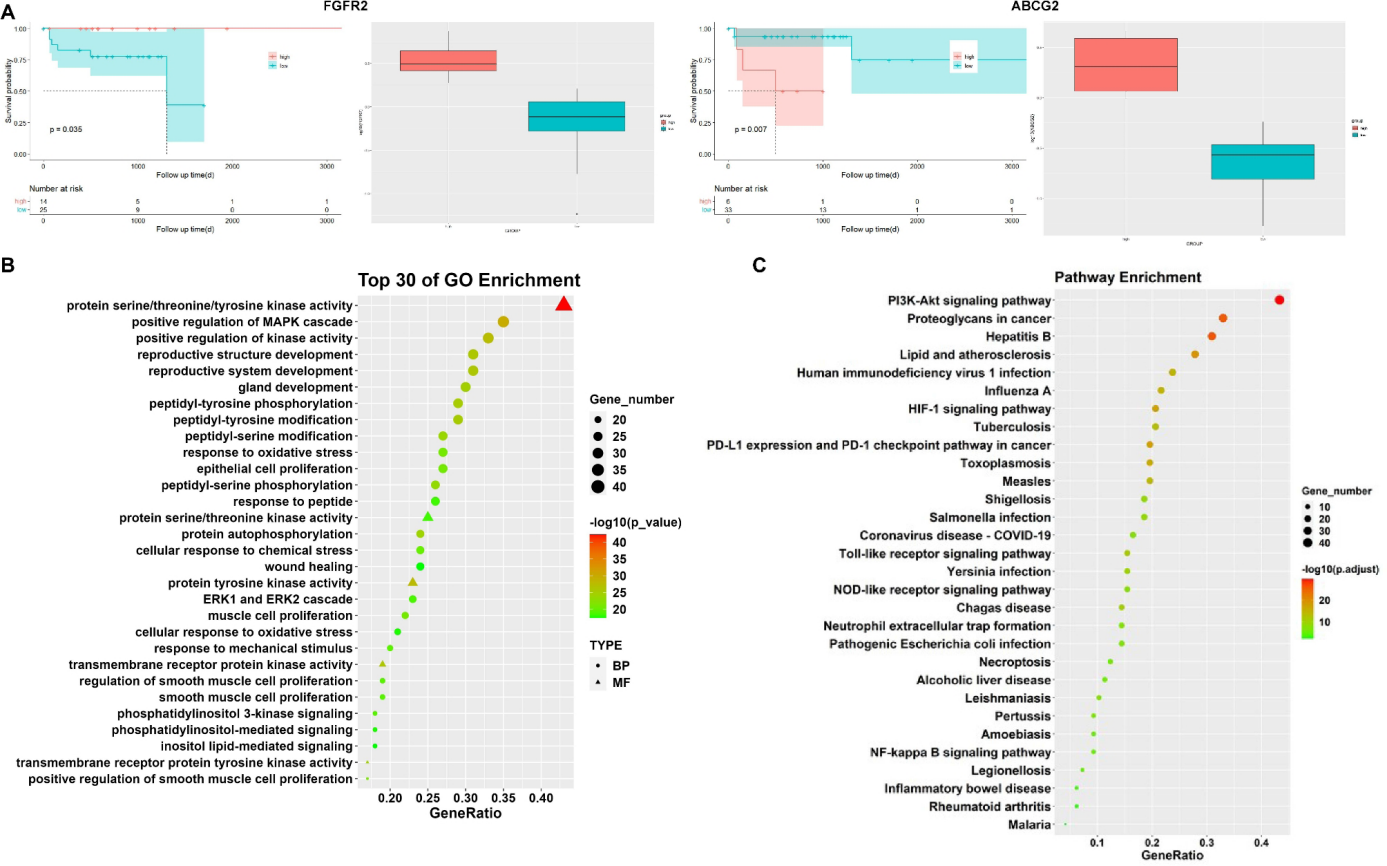
YGS and CRC co-target enrichment analysis. **A**, Survival analysis of differential genes. **B**, Bubble diagram of YGS and CRC co-target GO enrichment analysis; **C**, Bubble diagram of YGS and CRC co-target KEGG analysis

After the Venn diagram construction between the CRC differential genes via TCGA and the 100 action targets based on network pharmacology, nine overlapped genes ABCB1, ABCG2, BCL2, EGFR, ESR1, ESR2, FGFR2, PIK3CG, and VDR were identified. Survival analyses of these genes were performed, and only the difference between FGFR2 and ABCG2 was statistically significant (*P* < 0.01), as shown in Fig. 7A.

Figure 7B and C illustrate GO and KEGG analyses. Out of 100 overlapped targets, 4,335 GO entries were identified to be significant (*P* < 0.01). GO analysis results were partially displayed including the positive regulation of the Mitogen-Activated Protein Kinase (MAPK) cascade, protein serine/threonine kinase activity, membrane raft, membrane microdomain, transferase complex, and groups involved in phosphorus transfer. KEGG pathway enrichment analyses reveal 228 enriched pathways, which are partially depicted. Significant signaling pathways included the Phosphatidylinositol 3-Kinase - Protein Kinase B (PI3K-Akt) pathway, Hypoxia-Inducible Factor-1 (HIF-1) pathway, NF-κB pathway.

Based on the results in Sect. Screening of CRC key genes and Network pharmacology screening YGS affects candidate monomers target genes and pathway results of CRC stem cell metastasis relapse, YGS affected CRC candidate monomer was Gomisin B; the target gene was TLR4, and the action pathway was the NF-κB signaling pathway for subsequent research. Combined with the results described in Sect. Influences of YGS on metastatic relapse of CRC stem cells, we hypothesized that YGS might inhibit tumor immune escape by sensitizing CRC stem cells via the NF-κB signaling pathway by targeting TLR4, which was subsequently verified in vitro and in vivo.

### Verification of target genes and pathways affecting CRC stem cell metastasis relapse in in-vitro models

**Fig. 8:**
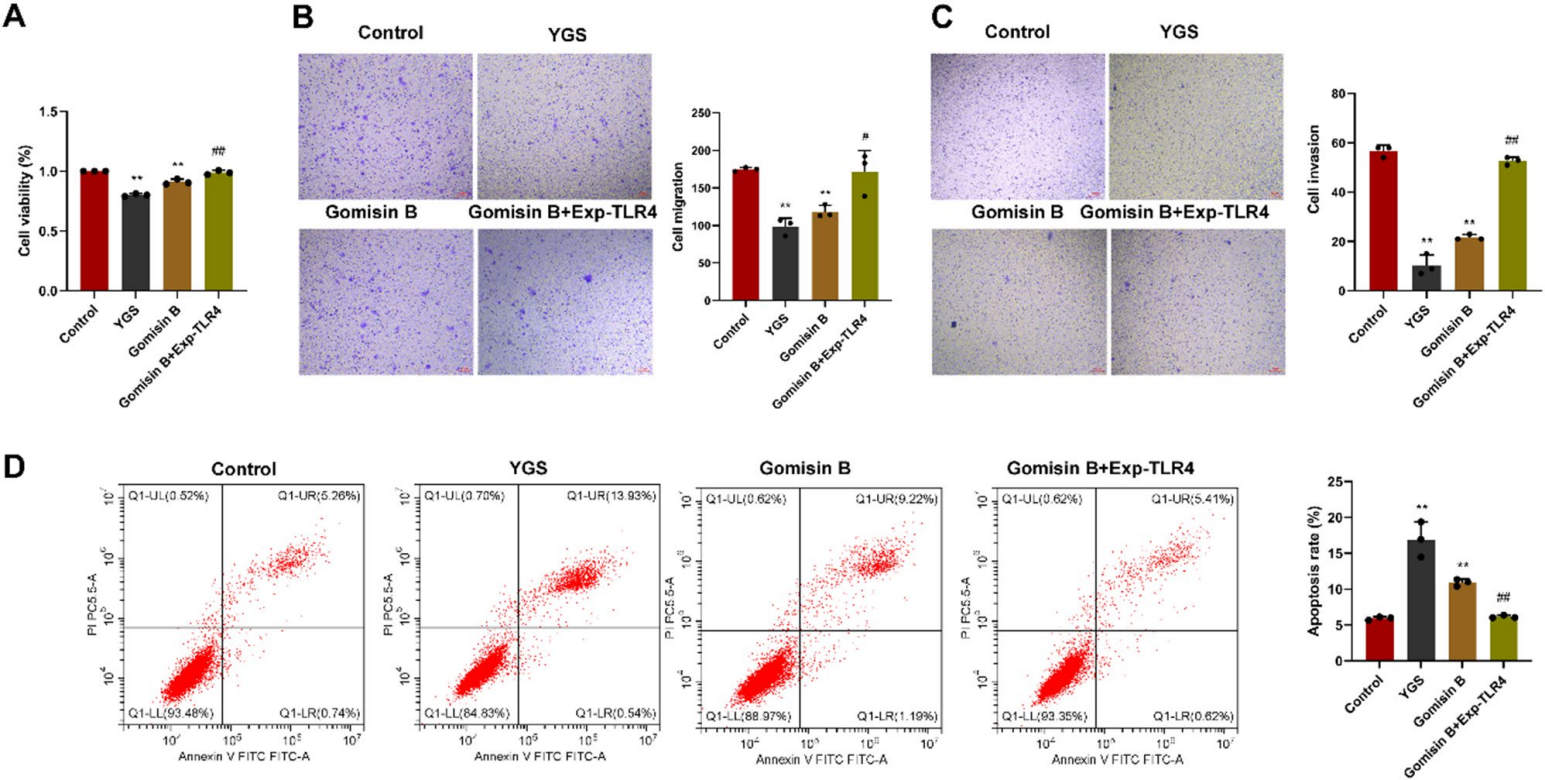
In-vitro models verification of YGS on the target genes and pathways of metastatic relapse of CRC stem cells. **A**, Impacts on cellular proliferation; **B**, Impacts on cellular migration; **C**, Impacts on cellular invasion; **D**, Effects on cell apoptosis. Exp-TLR4: overexpression of TLR4. Compared with the control group, * indicates *P* < 0.05, ** indicates *P* < 0.01; compared with the Gomisin B group, ^#^ indicates *P* < 0.05, ^##^ indicates *P* < 0.01, *n* = 3

Cell proliferation capacity was assessed using CCK-8 assays. The findings are displayed in Fig. 8A. In comparison to the CRC stem cell group, both the YGS and the Gomisin B groups exhibited a notable decrease in cell growth capacity. However, there were no notable alterations observed in the Gomisin B + TLR4 overexpression group. Scratch tests and Transwell invasion assays revealed that, when compared to the CRC stem cell group, YGS group and Gomisin B group had a significant reduction in cell migration and invasion (*P* < 0.01), while no substantial changes were noted in the Gomisin B + TLR4 overexpression group (Fig. 8B and C). Flow cytometry was employed to assess cell apoptosis (Fig. 8D). In contrast to CRC stem cell group, YGS and Gomisin B groups demonstrated an elevated level of apoptosis, with statistical significance (*P* < 0.01), whereas there was no substantial change observed in Gomisin B + TLR4 overexpression group.

**Fig. 9:**
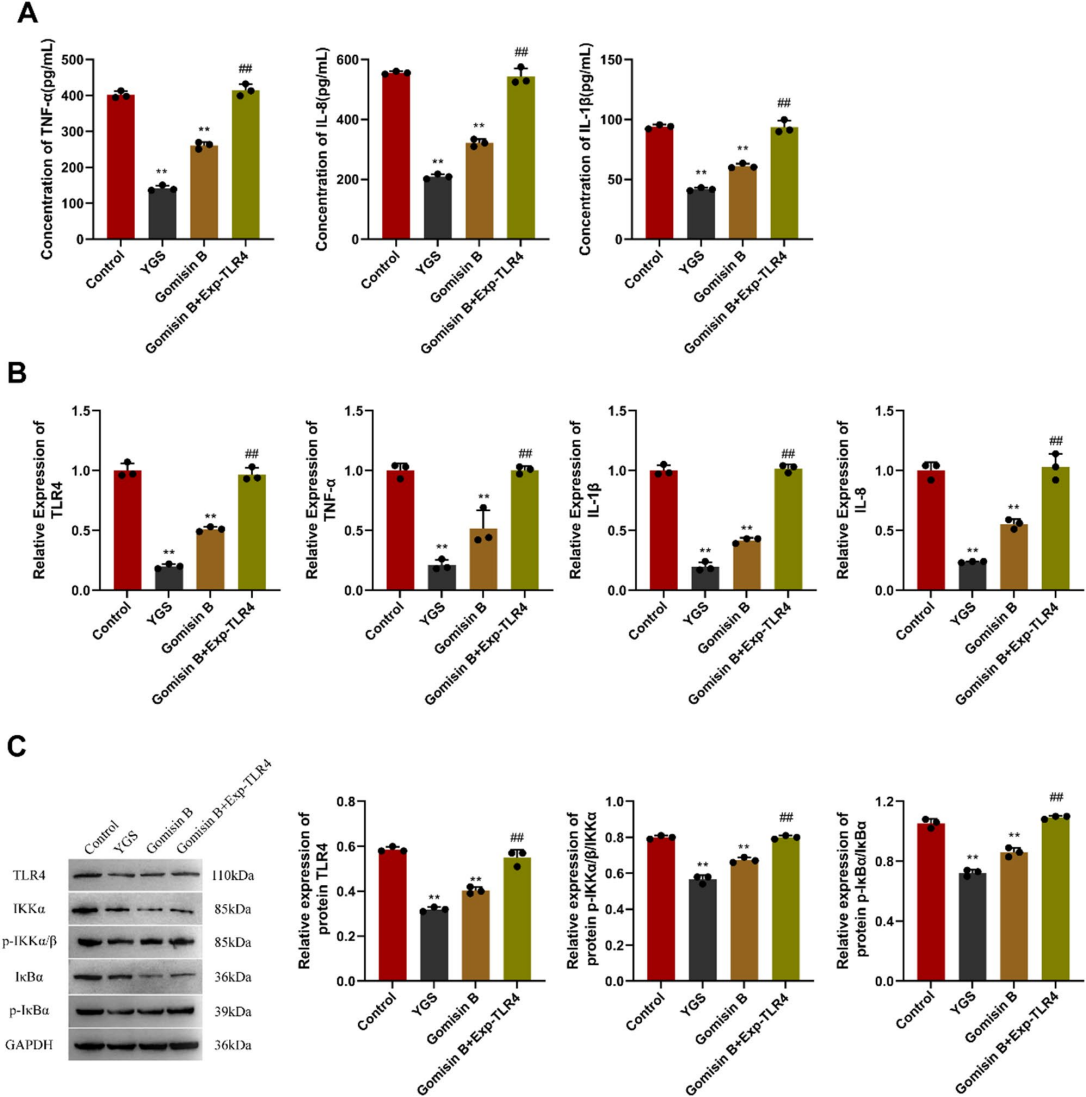
In-vitro models verification of YGS on the target genes and pathways of metastatic relapse of CRC stem cells. **A**, ELISA detection of cytokine expression levels; **B**, qPCR detection of TLR4 and cytokine transcription levels; **C**, WB detection of TLR4 and NF-κB pathway protein expression levels. Exp-TLR4: overexpression of TLR4. Compared with the control group, * indicates *P* < 0.05, ** indicates *P* < 0.01; compared with the Gomisin B group, ^#^ indicates *P* < 0.05, ^##^ indicates *P* < 0.01, *n* = 3

In Fig. 9A, ELISA assays were employed to determine cellular concentrations of TNF-α, IL-1β, and IL-8. By comparison, the concentrations of the three cytokines in the CRC stem cell group were higher than those in the YGS and Gomisin B groups, and there was a notable reduction in the latter two groups with statistical significance. However, there was no notable change in Gomisin B + TLR4 overexpression group. Figure 9B shows the results of qPCR detection, indicating that versus the CRC stem cell group, the relative expression levels of TLR4, TNF-α, IL-1β and IL-8 in the YGS group and the Gomisin B group were significantly reduced, with highly significant statistical differences (*P* < 0.01). Conversely, Gomisin B + TLR4 overexpression group exhibited no substantial alteration. The levels of TLR4 and NF-κB pathway proteins were detected by WB (Fig. 9C). Compared with the CRC stem cell group, the protein levels of TLR4, p-IKKα/β and p-IKBα in the YGS group and the Gomisin B group showed a statistically significant reduction (*P* < 0.01), while no obvious change was observed in the Gomisin B + TLR4 overexpression group.

Validation results in in-vitro models suggested that YGS sensitized CRC stem cells via the NF-κB signaling pathway by targeting TLR4 to inhibit tumor immune escape.

### Verification of target genes and pathways of YGS affecting metastatic relapse of CRC stem cells in vivo models

**Fig. 10:**
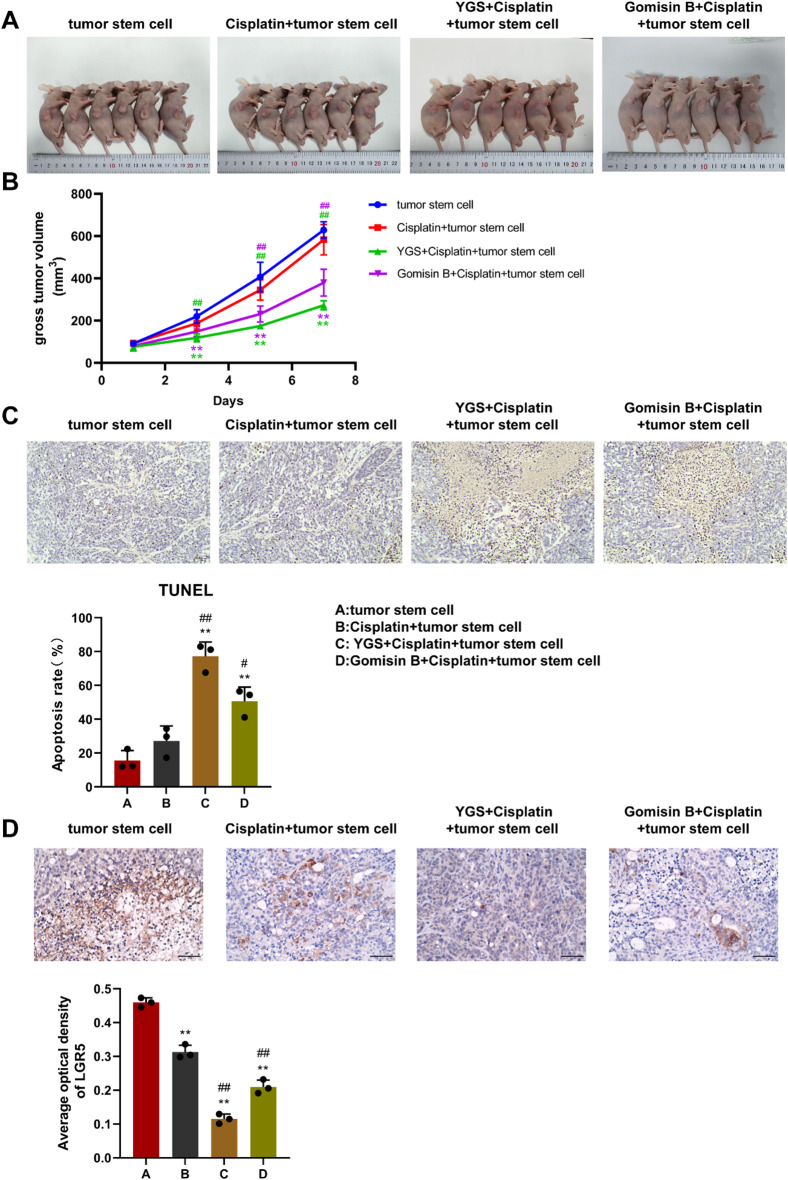
Verification in in vivo models of YGS affects target genes and pathways of metastatic relapse of CRC stem cells. **A**, Tumor growth images; **B**, Tumor growth curve; **C**, Detection of cell apoptosis by TUNEL. **D**, Immunohistochemistry detects the level of LGR5. Compared with the tumor stem cell group, * indicates *P* < 0.05, ** indicates *P* < 0.01; compared with the Cisplatin + tumor stem cell group, ^#^ indicates *P* < 0.05, ^##^ indicates *P* < 0.01, *n* = 3

As shown in Fig. 10A and B, the volume of tumors in the four groups increases as the treatment time increases. Compared with the CRC stem cell group, tumors in the cisplatin + stem cell group grew slowly, followed by the Gomisin B + cisplatin + stem cell group, and the YGS + cisplatin + stem cell group had the slowest growth rate (*P* < 0.01). Apoptosis was detected by TUNEL staining (Fig. 10C). Versus the CRC stem cell group, apoptosis in the YGS + cisplatin + stem cell group and the Gomisin B + cisplatin + stem cell group was elevated (*P* < 0.01), but there was no notable difference in the apoptosis of the cisplatin + stem cell group. Subsequently, immunohistochemical examination was carried out to detect the protein level of LGR5 in tumor tissues, as shown in Fig. 10D. Compared with the CRC stem cell group, the protein expression of LGR5 in the cisplatin + stem cell group, the YGS + cisplatin + stem cell group and the Gomisin B + cisplatin + stem cell group was markedly decreased (*P* < 0.01).

**Fig. 11:**
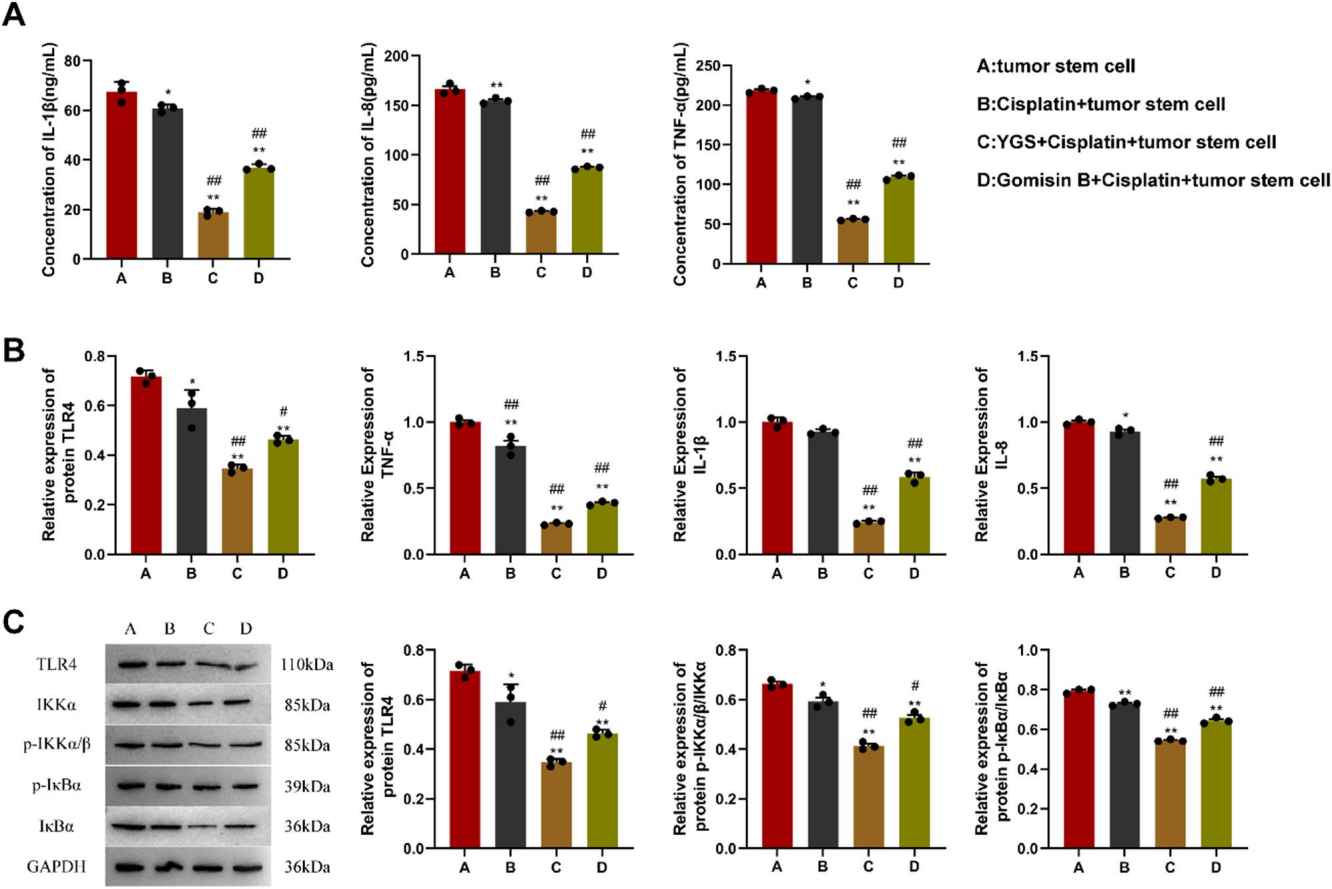
Verification in in vivo models of YGS affects target genes and pathways of metastatic relapse of CRC stem cells. **A**, ELISA detection of tumor tissue inflammatory factor levels; **B**, qPCR detection of tumor tissue target gene and inflammatory factor transcription levels; **C**. WB is used to detect the expression of tumor tissue target gene and NF-κB pathway protein. Compared with the tumor stem cell group, * indicates *P* < 0.05, ** indicates *P* < 0.01; compared with the Cisplatin + tumor stem cell group, ^#^ indicates *P* < 0.05, ^##^ indicates *P* < 0.01, *n* = 3

ELISA was employed to measure the concentrations of inflammatory cytokines within tumor tissue cells (Fig. 11A). In comparison to the CRC stem cell group, there was a noteworthy reduction in the levels of inflammatory factors in the cisplatin + stem cell group cells, with statistical significance (*P* < 0.05). Furthermore, YGS + cisplatin + stem cell group and Gomisin B + cisplatin + stem cell group displayed a remarkable decline in the inflammatory factor levels, with a highly significant difference (*P* < 0.01). We used qPCR assays to detect the transcription levels of tumor tissue target genes and inflammatory factors (Fig. 11B). Compared with the CRC stem cell group, the relative expressions of TLR4, TNF-α, IL-1β, and IL-8 in the cisplatin + stem cell group were markedly decreased. In addition, the relative expression levels of target genes and inflammatory factors in the YGS + cisplatin + stem cell group and the Gomisin B + cisplatin + stem cell group were markedly decreased. As shown in Fig. 11C, the expression of tumor tissue target genes and NF-κB pathway proteins were detected using WB. Compared with the CRC stem cell group, the protein levels of TLR4, p-IKKα/β, and p-IKBα in the cisplatin + stem cell group were markedly decreased. In addition, the protein levels of TLR4, p-IKKα/β, and p-IKBα in the YGS + cisplatin + stem cell group and the Gomisin B + cisplatin + stem cell group were markedly decreased.

**Fig. 12:**
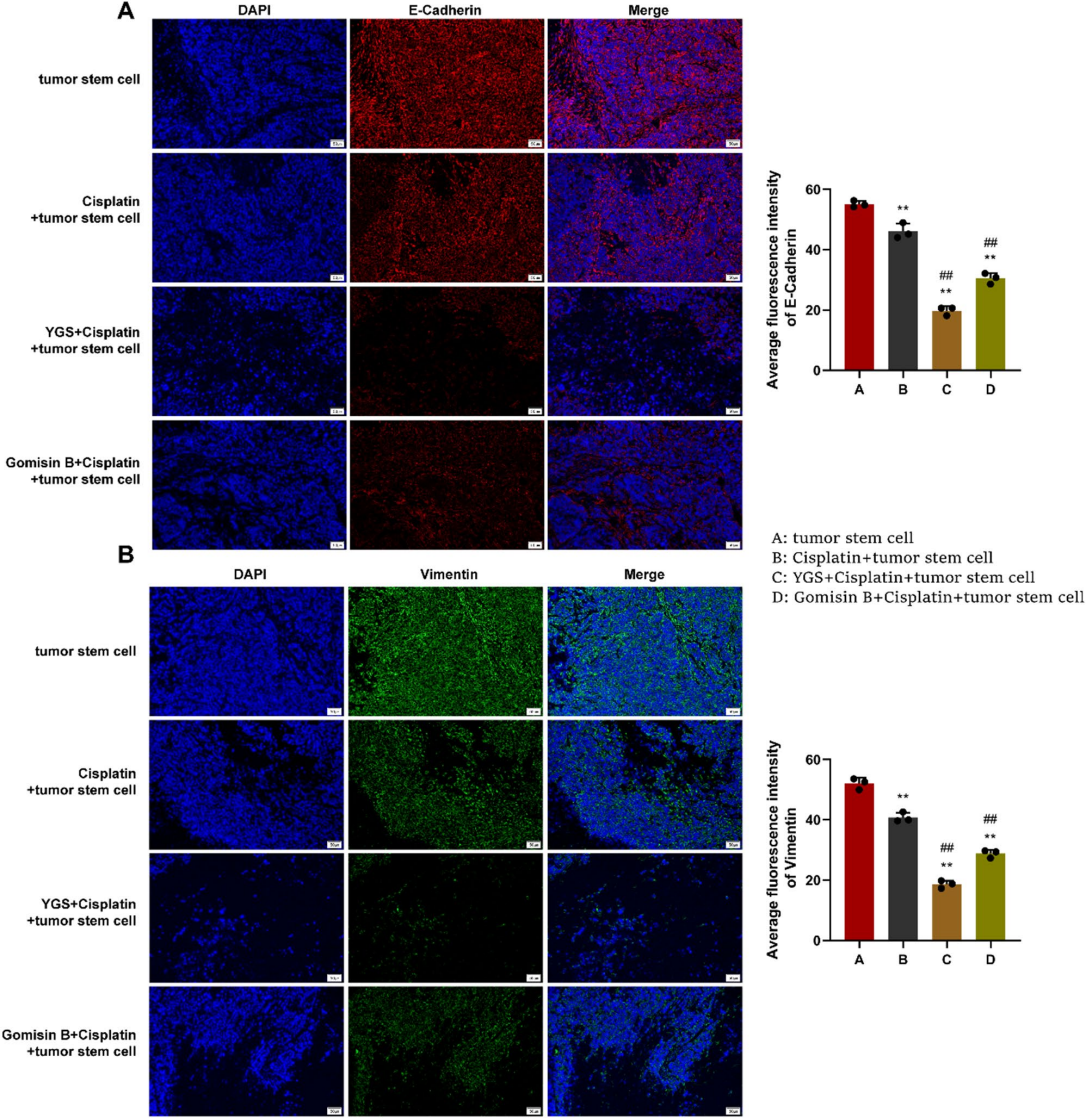
Immunofluorescence detects the expression of EMT markers in tumor tissues. **A**, E-Cadherin expression; **B**, Vimentin expression. Compared with the tumor stem cell group, * indicates *P* < 0.05, ** indicates *P* < 0.01; compared with the Cisplatin + tumor stem cell group, ^#^ indicates *P* < 0.05, ^##^ indicates *P* < 0.01, *n* = 3

Subsequently, immunofluorescence assay was performed to detect the protein expression of EMT markers E-Cadherin and Vimentin in tumor tissues (Fig. 12). Versus the CRC stem cell group, the protein levels of two markers in the cisplatin + stem cell group, the YGS + cisplatin + stem cell group and the Gomisin B + cisplatin + stem cell group was significantly reduced (*P* < 0.01).

The results in in-vivo models confirmed the hypothesis that YGS might inhibit tumor immune escape by sensitizing CRC stem cells via the NF-κB pathway through targeting TLR4, and YGS could inhibit the EMT process of CRC at the tissue level.

## Discussion

The United States sees CRC as the second most common cause of death due to cancer [21], and in China, CRC is also one of the most common malignancies. Despite the development of society and improvements in living standards, there have been a rapid increase in the morbidity and mortality rates of CRC [22]. A significant reduction in the rate of adverse reactions associated with YGS against CRC has been achieved, while limited research has been conducted on the mechanism of YGS against CRC. This study addressed the gap by focusing on the exploration of the YGS mechanism related to CRC treatment.

CRC stem cells were first isolated from SW480 colorectal cancer cells using a sorting method with magnetic beads [22]. ALDH has been confirmed as an indicator of stem cells [23], while SOX-2 expression stabilizes pluripotency in embryonic stem cells, embryonic pluripotent stem cells, and other early pluripotent cells [24]. Meanwhile, LGR5 is considered an important marker for CRC stem cells and is of great significance in the recognition and study of these cells [25]. Consequently, we used WB to detect the expression of ALDH, SOX-2 and LGR5 in stem cells, thereby confirming the identity of these stem cells as CRC stem cells. The effect of different concentrations of YGS on the nude mouse model was explored by constructing an animal model of CRC graft tumors. With increasing concentrations of YGS, the ability of tissue cells to proliferate, migrate and invade gradually decreased, while the percentage of apoptosis gradually increased, and the contents of TNF-α, IL-8 and IL-1β in the cell supernatant gradually decreased. The above results demonstrated that YGS could effectively inhibit immune escape of CRC stem cells. This result is consistent with the findings of researchers Song Ju Cai et al. [26], Yuan [27], and Liang Wanting et al. [28].

Network pharmacology is commonly employed to elucidate functional mechanisms of traditional Chinese medicine compounds. The present study utilized this approach to identify potential targets, candidate genes, and signaling pathways associated with CRC stem cell metastasis relapse with YGS treatment. Gomisin B was identified as the YGS candidate monomer through the TCMSP database, and TLR4 was identified as the target gene via the PPI network [29]. GO and KEGG analyses revealed that the enrichment of target genes occurred in pathways, including but not limited to the PI3K-Akt pathway, HIF-1 pathway, NF-κB pathway [30]. Therefore, the NF-κB pathway was selected for further investigation, hypothesizing that YGS might inhibit tumor immune escape by enhancing the sensitivity of CRC stem cells through the NF-κB pathway.

In comparison to the control group, YGS treatment group and Gomisin B treatment group markedly decreased the expression of the TLR4, p-IKKα/β, and p-IKBα proteins, with a statistically significant difference. However, there was no notable modification of protein expression in Gomisin B + TLR4 overexpression treatment group, which is consistent with the results of Jin Shaoju [31] and Xiang et al. [32]. Evidently, YGS, along with its constituent Gomisin B, could effectively impede the proliferation, migration, and invasion of CRC stem cells while promote the apoptosis of tumor cells. This resulted in a decrease in intracellular inflammatory factor content and a diminishment in the level of proteins within the NF-κB pathway, ultimately hampering the tumorigenesis and disease progression. However, when the target gene TLR4 was overexpressed, YGS inhibited the mechanism of CRC immune escape. It was feasible to select TLR4 as the target gene based on the study results and verify the reliability of the hypothesis.

Following an extended duration of detection, a notable increase was observed in tumor volume across the CRC stem cell group, cisplatin treatment group, YGS + cisplatin treatment group, and Gomisin B + cisplatin treatment group. The cisplatin, Gomisin B + cisplatin and YGS + cisplatin could inhibit the growth rate of nude mice. Moreover, the Gomisin B + cisplatin group and the YGS + cisplatin group could promote tumor tissue apoptosis and reduce the levels of inflammatory factors TNF-α, IL-1β, and IL-8. TLR4, p-IKKα/β, and p-IKBα protein levels in tumor tissues were markedly decreased in the YGS + cisplatin treatment group and in the Gomisin B + cisplatin treatment group (*P* < 0.01). The findings are consistent with those of Zeng [33], Chen [34], Huang [35] and Wan et al. [36]. Compared with the CRC stem cell group, immunofluorescence indicated a reduction in the level of EMT markers in neoplastic tissues of cisplatin treatment group, YGS + cisplatin treatment group and Gomisin B + cisplatin treatment group. This result is consistent with Xia [37] and Liu Leilei et al. [38].

The validation of in-vivo models aligned with the findings of in-vitro models, supporting the exploration of YGS’s inhibition of tumor immune escape through NF-κB pathway sensitization in CRC stem cells, and its capability to suppress the EMT process in CRC at the tissue level.

Currently, the treatment methods for CRC include surgery, chemotherapy, radiotherapy, and targeted therapy [39]. However, they are faced with challenges such as drug resistance, side effects, and immune escape. This study demonstrated that the combined treatment of YGS and cisplatin could enhance the anti-tumor effect of cisplatin, increase the sensitivity to chemotherapeutic drugs, and alleviate immune escape. In addition, YGS may enhance the immune response, inhibit EMT, and synergize with radiotherapy to improve radiosensitivity by regulating the NF-κB signaling pathway. Therefore, the combined use of YGS with traditional treatments is expected to overcome the limitations of single treatment and improve the therapeutic effect of CRC. Future research is needed to verify its potential for clinical application.

## Conclusion

YGS at different concentrations produced inhibitory effects on CRC cell proliferation, migration and invasion, and it enhanced apoptosis and suppressed inflammatory factor expression. The TCGA database and network pharmacology analyses identified the YGS candidate Gomisin B, the action target gene TLR4, and the action pathway NF-κB. YGS might inhibit tumor immune escape by sensitizing CRC stem cells through the NF-κB pathway, which was confirmed in vivo models and in vitro models.

## Electronic supplementary material

Below is the link to the electronic supplementary material.

Supplementary Material 1

## Data Availability

The original contributions presented in the study are included in the article.
